# The Association of Trp64Arg Polymorphism in the Beta-Adrenergic Receptor With Insulin Resistance: Meta-Analysis

**DOI:** 10.3389/fendo.2021.708139

**Published:** 2021-08-26

**Authors:** Hai-Dan Wang, Cai-Shun Zhang, Man-Wen Li, Qian Lin, Qing Zhang, De-Feng Liu, Zheng-Ye Ma, Jing Dong

**Affiliations:** ^1^Special Medicine Department, Medical College, Qingdao University, Qingdao, China; ^2^Clinical Medicine Department, Medical College, Qingdao University, Qingdao, China; ^3^Physiology Department, Medical College, Qingdao University, Qingdao, China

**Keywords:** Trp64Arg, insulin resistance, β3-adrenergic receptor gene, meta-analysis, subgroup analysis

## Abstract

**Background:**

Insulin resistance is a metabolic disorder that occurs in type 2 diabetes mellitus and obesity. Genetic factors such as β3-adrenoceptor polymorphism (Trp64Arg) may be involved in IR and insulin secretion. However, their association is controversial. Therefore, the current meta-analysis was conducted to clarify the relationship between the Trp64Arg and IR.

**Methods:**

The literature search was performed in PubMed, Embase, and Web of Science using the keywords “Receptors, Adrenergic, beta-3, Receptors, Adrenergic, Insulin Resistance, Protein-Coupled Receptor Kinase 3” from 2005 to February 7, 2021. We used a random-effects model to calculate the pooled effect size. We conducted subgroup analysis and regression analysis to identify sources of heterogeneity; and Egger’s test and funnel plot were used to test publication bias. Finally, we conducted a sensitivity analysis.

**Results:**

We included eight papers with 1,586 subjects. There was a positive correlation between Trp64Arg mutation and insulin level (standardized mean difference = 0.20, 95% confidence intervals: 0.00 to 0.39, *I* ^2^ = 57.6%, *p* = 0.016). However, there was no association between Trp64Arg and the homeostasis model (HOMA-IR) assessment. Egger’s tests showed no publication bias; the sensitivity analysis showed that our results were stable. Regression analysis revealed no source of heterogeneity.

**Conclusion:**

Trp64Arg may be associated with IR. European ancestry, obesity, plasma insulin level, and test status may be potential factors affecting the relationship between Trp64Arg and IR.

## Introduction

Insulin resistance (IR) is a metabolic disorder that can lead to type 2 diabetes and obesity (1). It can occur in the liver, white adipose tissues, and skeletal muscle (2–4). In clinical practice and epidemiological studies, fasting insulin levels and homeostasis model assessment (HOMA-IR) are commonly used substitute indices of IR (5). There is evidence to suggest that genetic factors are involved in IR and insulin secretion (6).

β-Adrenoceptors are the targets of endogenous catecholamines noradrenaline and adrenaline (7). β3-Adrenergic receptor gene (ADRB3) is a member of the β-adrenoceptor system (7) and plays an essential role in metabolic disorders (8). ADRB3 is expressed in human visceral adipose and is associated with increased lipolysis. In turn, increased lipolysis can lead to skeletal muscle IR (9). ADRB3 dysfunction may lead to IR and obesity and may be a candidate gene for obesity and IR (5). Winden et al. (10) reported that the ADRB3 polymorphism is the replacement of tryptophan (Trp64Trp) by arginine at position 64 (Trp64Arg) of the β3-adrenergic receptor. Studies showed that the Trp64Arg mutation was associated with type 2 diabetes mellitus, IR, and body weight increases (11, 12). Subsequently, many studies investigated the relationship between this common variant and various metabolic syndrome phenotypes, including elevated body mass index

(BMI); Trp64Arg mutation had a specific effect on BMI (13–15). Similarly, the relationship between IR or its related indices and Trp64Arg was also studied. In 2005, a meta-analysis indicated that the Trp64Arg variant was associated with IR (5). However, studies after 2005 remained controversial, and there has been no meta-analysis to clarify the relationship between the Trp64Arg and IR. For example, Højlund et al. (16) demonstrated that Trp64Arg might not increase IR, and another study suggested that Trp64Arg had no relationship with IR (17). However, another study showed that a mutation group of patients with Trp64Arg had higher insulin and HOMA-IR levels (18).

Therefore, to help resolve the discrepancies among these studies, we analyzed the association between Trp64Arg and IR or its related indices in this meta-analysis.

## Research Methods

### Search Strategy

**Figure 1:**
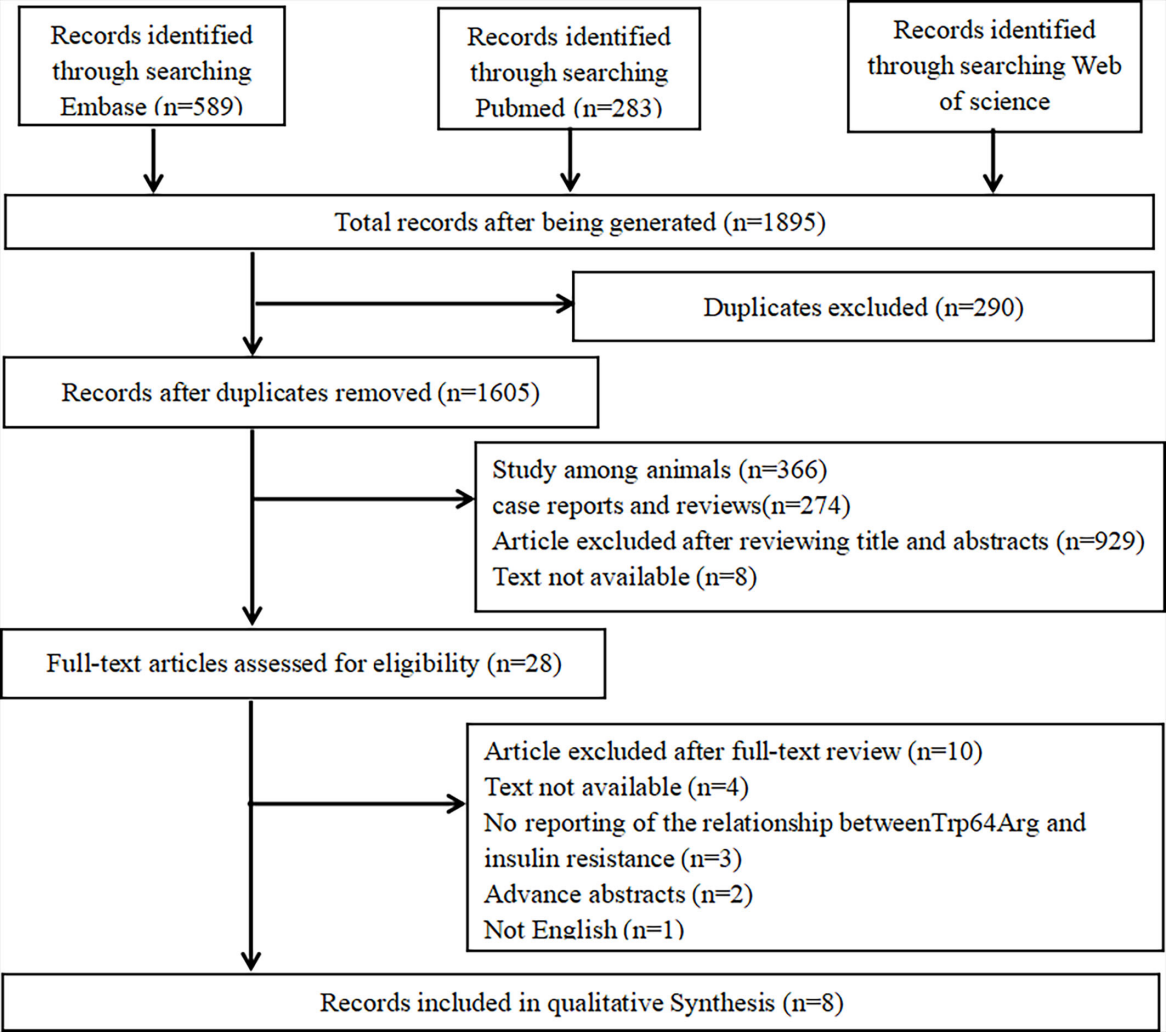
Flow chart of the included studies.

We performed the meta-analysis based on Preferred Reporting Items for Systematic Reviews and Meta-Analyses guidelines (19). A literature research was performed in PubMed, Web of Science, and Embase. Two groups of keywords and their major subheading terms were used to search relevant studies including receptors, adrenergic, beta-3 (e.g., “beta-3 Adrenergic Receptors,” “Receptors, Adrenergic,” “G-Protein-Coupled Receptor Kinase 3,” “TRP64Arg,” “TRP64Arg polymorphisms,” “ADRB3,” “adrenergic receptor gene,” “adrenergic receptor gene,” and “β3-AR”) and IR (e.g., “Resistance, insulin,” “Insulin Sensitivity,” and “Sensitivity, Insulin”). The date range was from January 1, 2005 to February 7, 2021. More details are shown in **Figure 1**.

### Inclusion and Exclusion Criteria

The inclusion criteria were as follows: 1) studies of humans; 2) inclusion of means, the standard deviation (SD) of related indices of IR, including insulin level and HOMA-IR by genotypes and by subgroups; 3) publication in English; and 4) observational studies with subjects ≥10. The exclusion criteria were as follows: 1) case reports, reviews, comments, protocols, meeting abstracts, and meta-analyses; 2) no study of the correlation between Trp64Arg and IR; 3) other interventions; and 4) inability to assess the full text.

### Data Extraction and Quality Assessment

For data extraction, two independent reviewers reviewed all studies and extracted the data in a standardized format. The data were collected after all the disagreements were resolved. The collected information included the first author of the studies, the year of publication, country, sample size, the number of men and women, and the effect value. Subjects’ characteristics such as age, BMI, health condition, and test status of insulin were also extracted as potential variables for further subgroup analysis. To unify the units of insulin, the conversion factor 6.945 (1 mU/L = 6.945 pM) was applied to transform insulin concentration from picomolar into milliunits per liter (20). The qualification of the cross-sectional studies was based on Agency for Healthcare Research and Quality (21), and Newcastle-Ottawa Scale was used to assess the qualifications of case–control studies. All assessments were performed by two authors independently. Specific criteria and scores are presented in **Supplementary Table 1**.

### Statistical Analysis

The associations between Trp64Arg and IR were analyzed using the random-effects model, which used the mean values and their SD. Forest plots were also drawn. *I* ^2^ was used to show the heterogeneity; *I* ^2^ ≥50% and *p*-value <0.05 indicated significant heterogeneity (22). If the results showed heterogeneity, meta-regression analysis was used to explore its sources (23). We conducted influence analysis to determine the impact of a single study on the overall results. If the results showed that one study impacted on the overall results, we conducted a sensitivity analysis to determine the stability of the results. Cumulative analysis was performed to evaluate the trend of pooled effect estimates over time. Publication bias was analyzed using funnel plots and Egger’s tests. We also performed subgroup analysis based on age, country, BMI, blood samples, and test status. All the analyses were performed using Stata software 12.0.

## Results

### Literature Search

Search details are shown in **Table 1**. We searched PubMed, Web of Science, and Embase. In total, 1,895 potential studies were filtered from online databases, and 290 studies were removed because of duplication, 1,577 ineligible studies were removed after scanning the title and abstracts, and 20 were removed after scanning the full texts. Finally, eight studies met the selection criteria (24–31).

**Table 1:** Characteristics of included studies.

Author	Country	Study type	Test status	Number (Female/Male)	Age	BMI	Health condition	TA/TT (N)	Blood sample	Quality score
Pierola et al. (24)	Span	Case–control	Fasting	337/50	50 ± 11	31.9 ± 5.9	OSAS	71/316	Plasma	8
Pérez-Bravo et al. (25)	Chile	Case–control	Fasting	106	25.10 ± 5.64	26.65 ± 5.34	Normal	29/53	Serum	7
Pérez-Bravo et al. (25)	Chile	Case–control	Fasting	82	23.58 ± 5.19	29.11 ± 6.08	PCOS	43/63	Serum	8
Erhardt et al. (26)	Hungary	Case–control	75-g OGTT	127/168	12.6 ± 3.2	30.6 ± 4.7	Obese	35/35	Serum	8
de Luis et al. (27)	Span	Cross-sectional	Normal	170/94	41.1 ± 13.1	36.5 ± 5.9	Obese	38/226	Plasma	9
de Luis et al. (28)	Helsinki	Case–control	Normal	154/58	44.8 ± 16.7	35.8 ± 4.9	Obese	50/162	Serum	8
Dunajska et al. (29)	Poland	Case–control	Fasting	284	50-60	–	Postmenopausal	36/243	Serum	7
Mirrakhimov et al. (30)	Ethnic Kyrgyz	Cross-sectional	Fasting	145/68	50.7 ± 7.6	–	MS	46/80	Plasma	8
Zawodniak-Szalapska et al. (31)	Poland	Case–control	75-g OGTT	38/22	13.3 ± 2.94	–	Obese	14/46	Plasma	6

Data are presented as mean ± standard deviation.

TA, Trp64Arg; TT, Trp64Trp; BMI, body mass index; OSAS, obstructive sleep apnea syndrome; PCOS, polycystic ovary syndrome; MS, metabolic syndrome.

### Study Characteristics

All of the studies were published from 2005 to 2021 and included 362 subjects with the Trp64Arg variant and 1,224 subjects with normal genotype. The characteristics of the studies are presented in **Table 1**. Six case–control studies (24–26, 28, 29, 31) included 1,196 individuals and two cross-sectional studies (27, 30) that included 390 individuals. Four studies were conducted among obese populations (26–28, 31), one study was conducted among the normal (25) population, and the other four studies were conducted among people with obstructive sleep apnea syndrome (24), polycystic ovary syndrome (25), postmenopausal status (29), and metabolic syndrome (30). The age ranged from 13 to 60 years. More details are presented in **Table 1**.

### Overall Analysis

**Figure 2:**
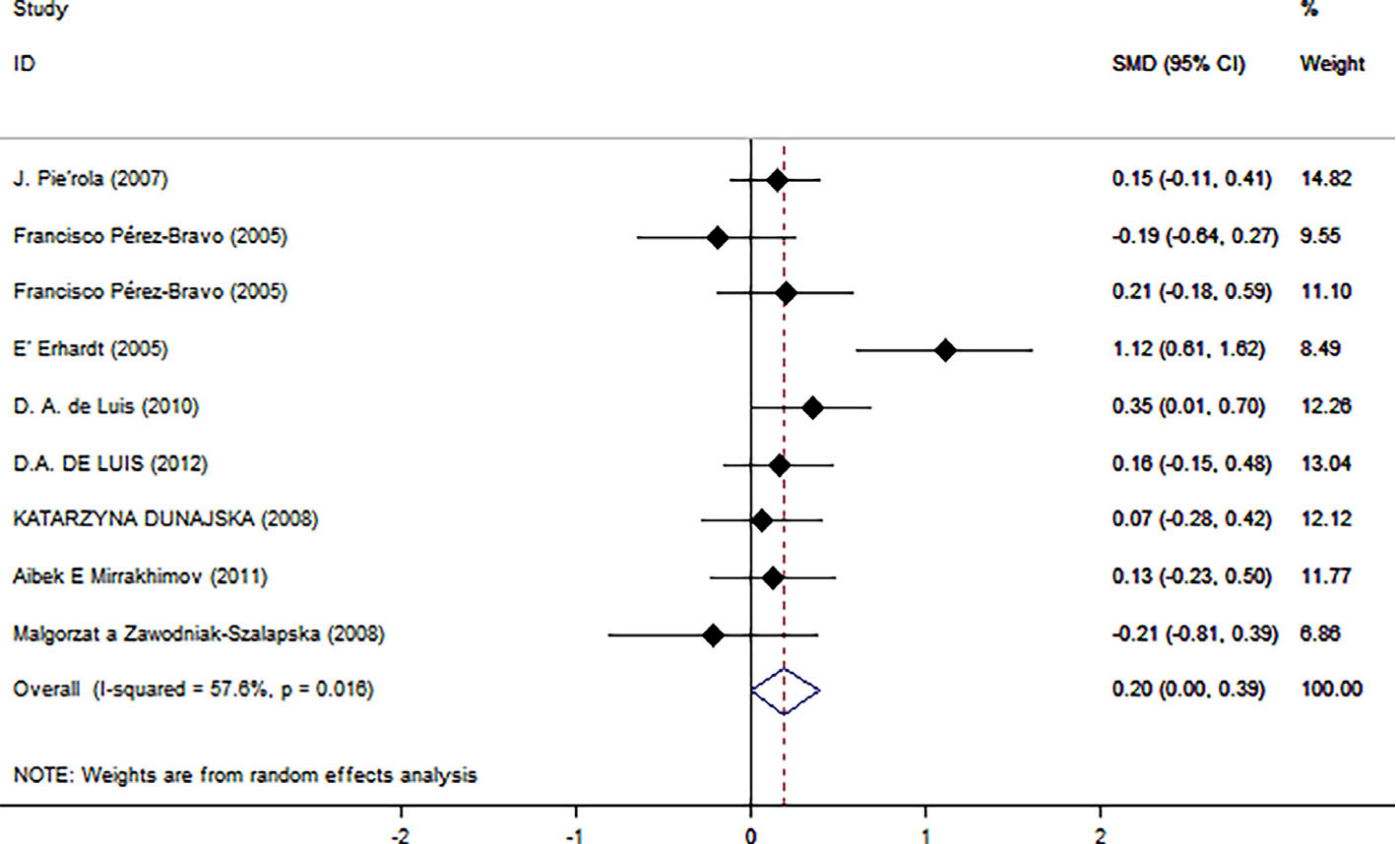
Insulin level as an index of insulin resistance. Forest plot showing the effect size of the correlation between Trp64Arg mutation and insulin resistance. 95% confidence intervals (CIs) are expressed in bars (each group) and diamond (all studies). Summary estimates are analyzed using a random-effects model. SMD, standardized mean difference.

**Figure 3:**
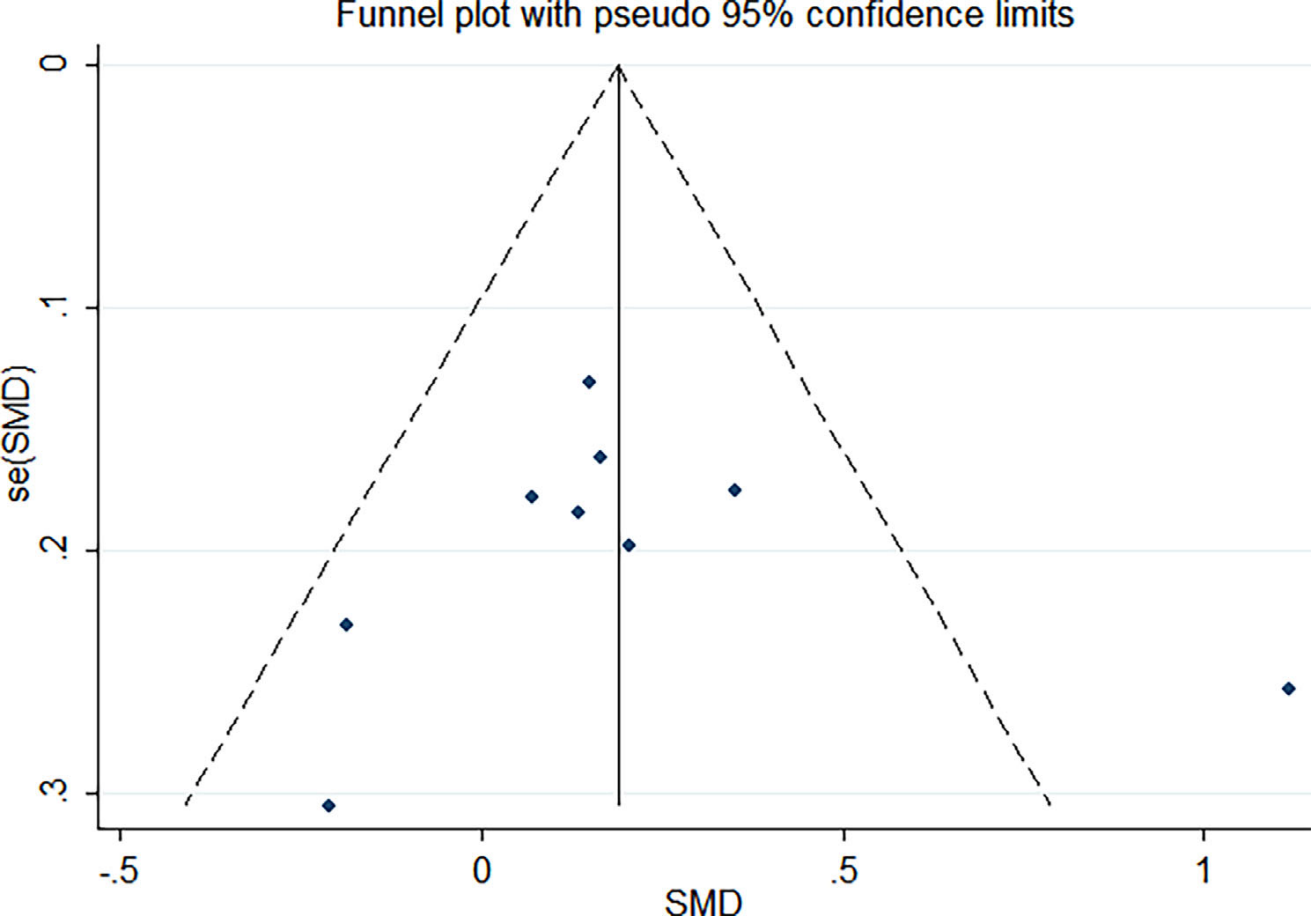
Funnel plot of the publication bias.

As shown in **Figure 2**, there was a positive correlation between insulin and Trp64Arg variant genotype (standardized mean difference (SMD) = 0.20, 95% confidence interval (CI): 0.00 to 0.39). There was heterogeneity among studies (*I* ^2^ = 57.6%, *p* = 0.016). In the influence analysis, we found that Erhardt’s study (7) had a greater impact on the stability of the combined result (**Supplementary Figure 1**). The sensitivity analysis showed that after excluding that study, the re-fitted result remained statistically significant, with an SMD and 95% CI of 0.13 (0.01, 0.26) (**Supplementary Figure 2**). Egger’s test and the funnel plot showed no publication bias (*p* = 0.796) (**Figure 3** and **Supplementary Figure 3**).

### Subgroup Analysis

**Figure 4:**
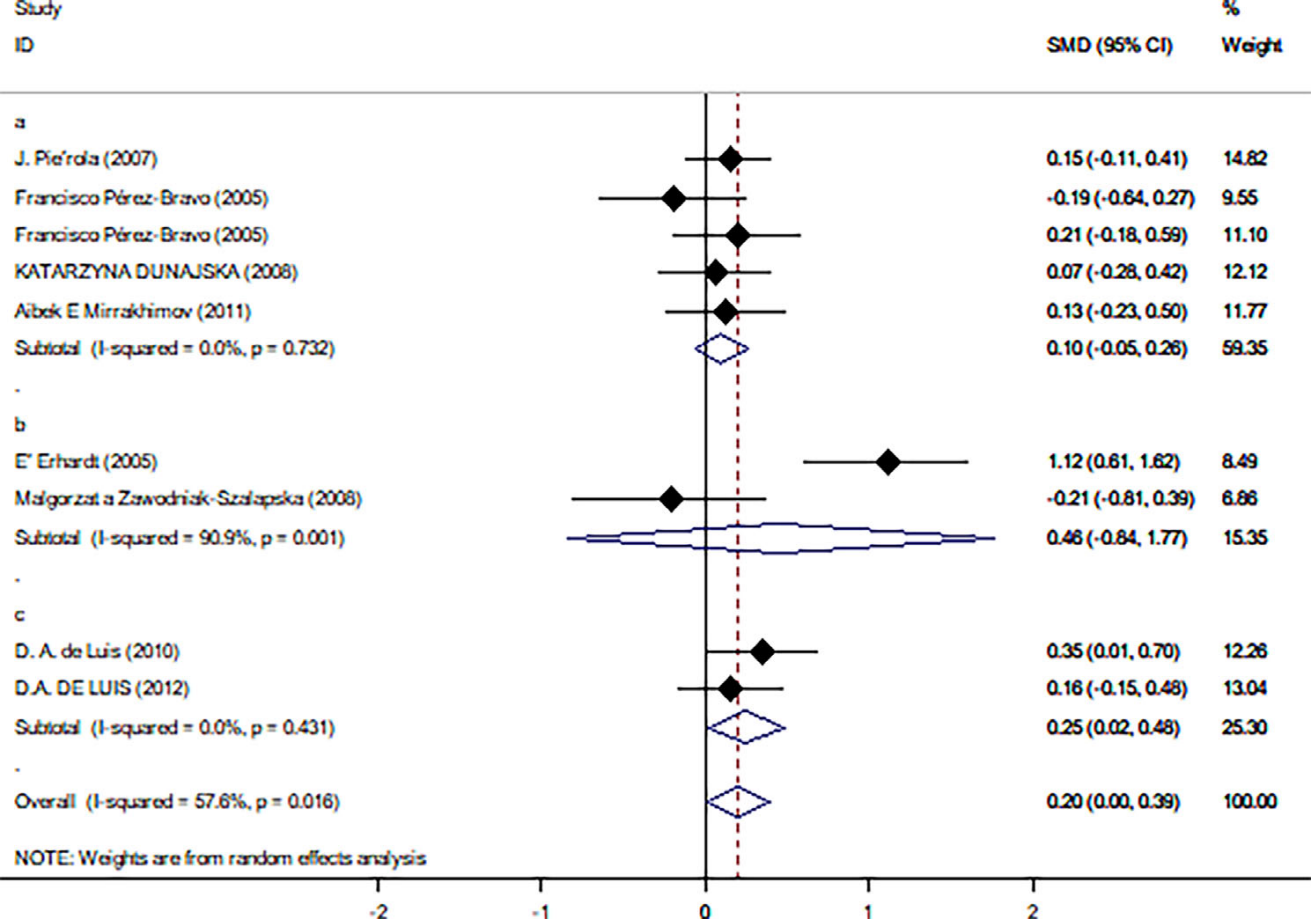
Forest plot of correlations between Trp64Arg mutation and insulin resistance-based onset status. 95% confidence intervals (*CI*) are expressed in bars (each group) and diamond (all studies). Summary estimates were analyzed using a random-effects model. Pierola et al., Pérez-Bravo et al., Dunajska et al., and Mirrakhimov et al. presented data from people with fasting test status; Erhardt et al. and Zawodniak-Szalapska et al. presented data from people with 75-g OGTT test status. de Luis et al. presented data from people with normal test status. SMD, standardized mean difference; OGTT, oral glucose tolerance test.

**Table 2** summarizes the results of the subgroup analysis of the relationship between Trp64Arg and insulin levels and HOMA-IR based on the participants’ characteristics. First, we found that the associations between Trp64Arg genotype and insulin levels differed depending on test status. Specifically, all subjects were divided into three groups: the fasting group, the 75-g oral glucose tolerance test (OGTT) (1.75 g/kg ideal body weight, max. 75 g) group, and the normal group. Studies conducted under the normal test status revealed a positive correlation between Trp64Arg mutation and insulin levels (SMD = 0.25, 95% CI: 0.02 to 0.48, *I* ^2^ = 0%, *p* = 0.431) (**Figure 4**). However, no association was found between Trp64Arg and insulin levels in the subgroup of fasting condition and 75-g OGTT condition. We also conducted subgroup analysis based on blood sample (plasma or serum), ethnicity (Asian, South American, and European), and BMI (overweight or obesity) (32). However, we did not find differences among these factors.

**Table 2:** Subgroups analyses of relationship between Trp64Arg mutation and insulin resistance.

	Groups	Participants (N)	Random-effects SMD (95% CI)	*I*^2^ (%)	*p* for heterogeneity
**Insulin**				57.6	
Overall	9	1,586	0.20		0.016
Subgroup analyses					
*Country*					
Asian	1	126	0.13 (−0.23, 0.5)	–	–
South America	2	188	0.03 (−0.35, 0.41)	39.6	0.198
Europe	6	1,272	0.26 (−0.00, 0.53)	68.3	0.007
*Age*					
<18	2	130	0.46 (−0.84, 1.77)	90.9	0.001
≥18	7	1,456	0.15 (0.02, 0.28)	0	0.713
*BMI*					
Overweight	4	593	0.07 (−0.12, 0.27)	0	0.612
Obesity	5	993	0.31 (−0.01, 0.63)	72.9	0.005
*Blood sample*					
Plasma	4	837	0.17 (−0.00, 0.34)	0	0.443
serum	5	749	0.25 (−0.10, 0.61)	75.1	0.003
Test status					
Normal	2	476	0.25 (0.02, 0.48)	0	0.431
75-g OGTT	2	130	0.46 (−0.84, 1.77)	90.9	0.001
Fasting	5	980	0.10 (−0.05, 0.26)	0	0.732
**HOMA**					
Overall	6	1,121	0.08 (−0.40, 0.55)	90.2	0
Subgroup analysis					
*Country*					
South America	2	188	−0.65 (−1.53, 0.23)	87.8	0.004
Europe	4	933	0.40 (0.05, 0.74)	75.4	0.007
*Age*					
<18	1	70	1.12 (0.61, 1.62)	–	–
≥18	5	1,051	−0.01 (−0.56, 0.34)	88.1	0
BMI					
Overweight	2	188	−0.65 (−1.53, 0.23)	87.8	0.004
Obesity	4	933	0.40 (0.05, 0.74)	75.4	0.007
*Blood sample*					
Plasma	2	651	0.22 (0.02, 0.43)	0	0.356
serum	4	470	−0.01 (−0.83, 0.81)	93.6	0
Test status					
Normal	2		0.25 (0.02, 0.48)	0	0.431
75-g OGTT	1		1.12 (0.61, 1.62)	–	–
Fasting	3		−0.37 (−1.12, 0.38)	91.9	0

Data presented as mean ± SD.

OGTT, oral glucose tolerance test; OSAS, obstructive sleep apnea syndrome; PCOS, polycystic ovary syndrome; MS, metabolic syndrome; TA, Trp64Arg; TT, Trp64Trp.

**Figure 5:**
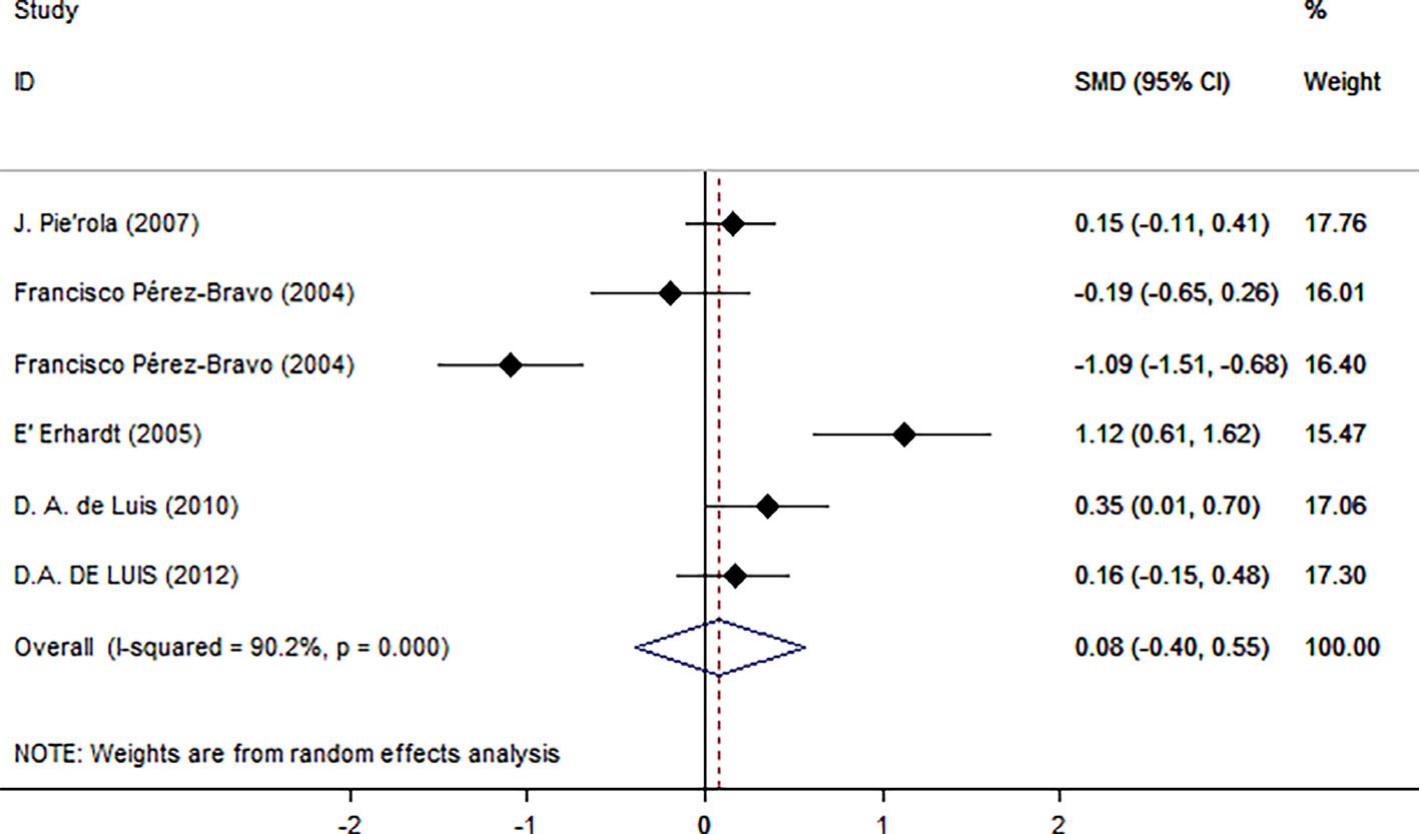
Using HOMA as an index of insulin resistance. Forest plot showing the effect size of the correlation between Trp64Arg mutation and insulin resistance. 95% confidence intervals (CIs) are expressed in bars (each group) and diamond (all studies). Summary estimates are analyzed using a random-effects model. SMD, standardized mean difference.

Next, we used HOMA-IR as an index of IR (**Figure 5**). A positive relationship was found only for normal test status (SMD = 0.25, 95% CI: 0.02 to 0.48, *I* ^2^ = 0%, *p* = 0.431), and not for other test status. We also found a positive relationship only in plasma blood samples (SMD = 0.22, 95% CI: 0.02 to 0.43, *I* ^2^ = 0%, *p* = 0.356), obese populations (SMD = 0.40, 95% CI: 0.05 to 0.74, *I* ^2^ = 75.4%, *p* = 0.007), and European ethnicity (SMD = 0.40, 95% CI: 0.05 to 0.74, *I* ^2^ = 75.4%, *p* = 0.007) (**Table 2**).

### Meta-Regression Analysis

To identify sources of heterogeneity, we conducted a meta-regression analysis. Covariates including age, country, blood sample, test status, and BMI were explored. None of these factors was the source of the heterogeneity (**Table 3**).

**Table 3:** Meta-regression of correlations between Trp64Arg and insulin resistance.

	Coefficient β	SE	95% CI	*p-*Value
Country	0.231	0.424	(−1.119, 1.580)	0.624
Test status	−0.066	0.307	(−1.042, 0.909)	0.842
BMI	−0.702	0.829	(−3.340, 1.937)	0.459
Blood sample	0.398	0.490	(−1.163, 1.959)	0.476
Age	−0.001	0.300	(−0.956, 0.954)	0.997

BMI, body mass index.

### Cumulative Analysis

**Figure 6:**
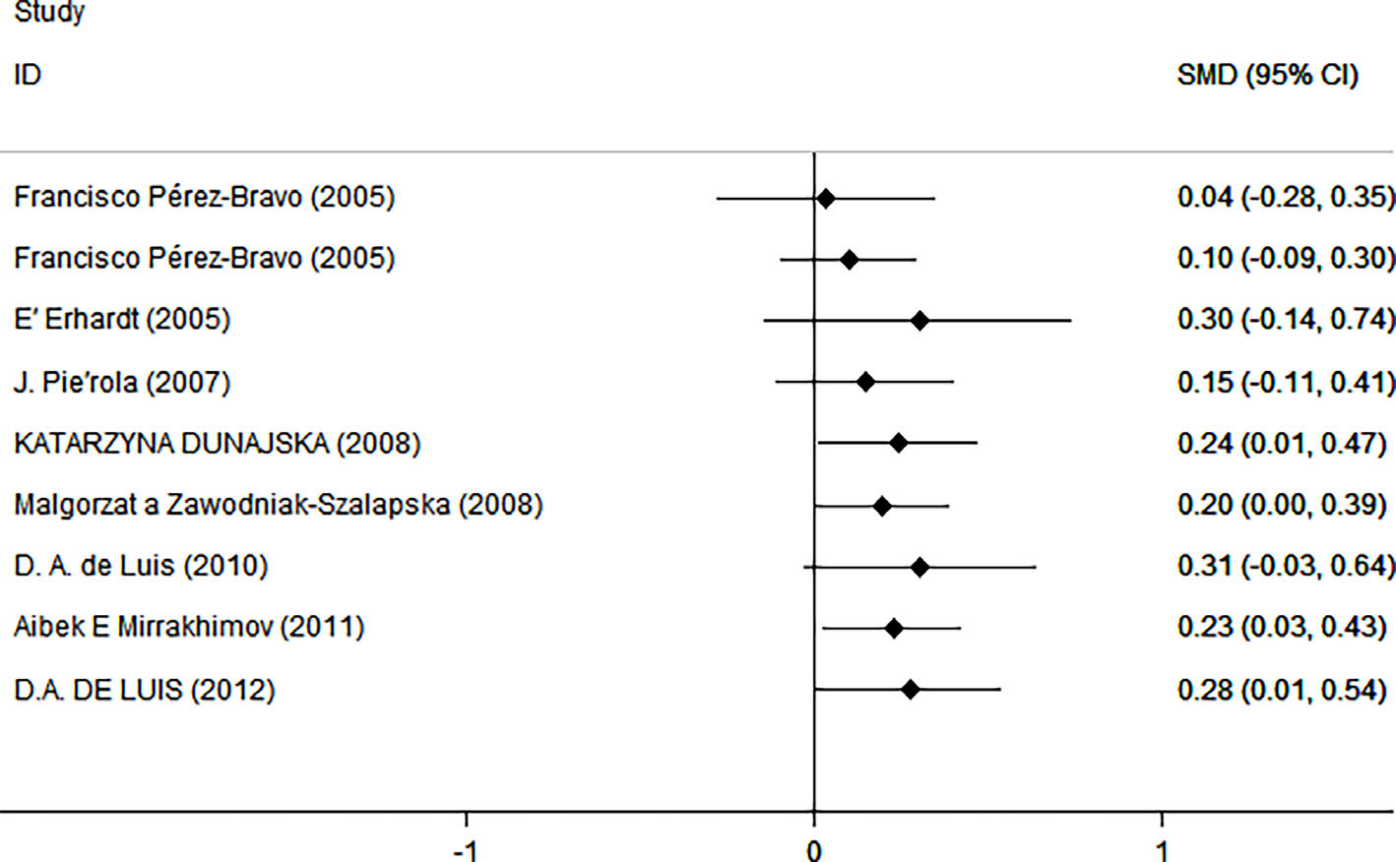
Cumulative analysis.

To evaluate the trend of pooled effect estimates over time, we did a cumulative analysis. The results showed that the pooled result was stable over time (**Figure 6**).

## Discussion

ADRB3 is an important regulator of many physiological functions, such as thermogenesis of brown adipose tissue and lipolysis of white adipose tissue. There are many variations in ADRB3 polymorphism; among these mutations, the most common ADRB3 mutation is Trp64Arg (33). Strosberg et al. mentioned that the allele Arg64 genotype is expressed in nearly all populations of the world (34); the allele can be activated by its agonists (33). Researches showed that ADRB3 polymorphic variants are associated with many diseases, such as cardiovascular diseases, obesity, diabetes, and other disorders (35). This provides insight into the potential pathophysiological effects of ADRB3 polymorphism in the development of these diseases.

We found that the Trp64Arg variant had a positive correlation with IR. The results of the cumulative analysis show that the pooled result was stable over time. Subgroup analysis suggested that test status was a factor that may influence the relationship between Trp64Arg and IR. When using HOMA-IR as an index of IR, subgroup analysis showed that the association between Trp64Arg and IR might be affected by the type of blood sample, obesity, and ethnicity. Heterogeneity in studies is expected in meta-analyses (36); exploring the potential sources of heterogeneity is an essential part of the analysis. What should be noted is that there was heterogeneity between studies in our meta-analysis. Therefore, we used meta-regression analysis to identify the covariates that may have a substantial impact on the heterogeneity, including age, ethnicity, test status, BMI, and source of a blood sample. However, none of these covariates affected the heterogeneity in the meta-regression analysis. Subsequently, considering the heterogeneity of the meta-analysis included in the study, we conducted subgroup analyses based on test status, blood sample source, ethnicity, and BMI.

Subgroup analyses suggested that test status might affect the relationship between the Trp64Arg variant and IR. Adeva-Andany et al. (37) mentioned in their review that dietary ingredients can affect the degree of insulin sensitivity; therefore, dietary ingredients under normal test status may influence the association between the Trp64Arg variant and IR. Some ingredients in the diet can affect IR; for example, Rivellese et al. mentioned that saturated fatty can increase IR; thus, subjects included in the researches may eat saturated fatty acids when they are under normal test status (38). In addition, the concentration of free fatty acids in type 2 diabetes or obese patients is increased; free fatty acids are a major factor contributing to IR (39). On the other hand, people with normal test status are characterized by obesity, which may be another cause of the differences (27, 28). Moreover, this finding can also support the conclusion that the effect mutation on IR may be affected by obesity (33). Papandreou et al. found that several plasma amino acids may be associated with IR, including branched-chain amino acids, aromatic amino acids, alanine, proline, and glutamine (40, 41). These findings may explain the potential influence of blood samples on the relationship. The allele frequency of Trp64Arg varies among different ethnicities (42). These findings suggest that ethnicity may influence the relationship between the Trp64Arg variant and IR.

In the influence analysis, we found that Erhardt’s study had a more significant impact on the pooled results, which may affect the stability of the results (**Supplementary Figure 1**). After this study was excluded, the results remained statistically significant, suggesting that the results were stable (**Supplementary Figure 2**). Egger’s test and funnel plots showed that there was no publication bias.

A previous meta-analysis showed that Trp64Arg had a small association with BMI (43). When using HOMA-IR as an index of IR, BMI affected the relationship between Trp64Arg and IR. Considering that IR is associated with obesity (44), further subgroup analysis indicated that the association between Trp64Arg and IR was significant in obesity. Therefore, we speculate that obesity may be the cause of the Trp64Arg mutation that leads to IR. Garcia-Rubi et al. (45) found that obese women with Trp64Arg mutation had greater IR than had women who carried Trp64Arg genotype based on age, body composition, and physical activity. However, Urhammer et al. (46) found that Trp64Arg may be associated with IR and was not affected by BMI. If the relationship between IR and Trp64Arg mutation can be attributed to obesity, we should pay attention to the underlying mechanism. The Trp64Arg variant has been shown to reduce lipolysis (47). Another study found that the Trp64Arg mutation can influence body weight loss (48). These findings suggest that the underlying mechanism may be related to impaired lipolysis, which leads to the increased adipocytes and then obesity, which may lead to IR and hyperinsulinemia.

In addition to IR, the Trp64Arg mutation is associated with insulin secretion. In a study of male twins who carried the Trp64Arg mutation, IR and insulin secretion (determined by homeostasis model assessment) were significantly lower (49). Furthermore, Christiansen et al. demonstrated that Trp64Arg polymorphism had no effects on plasma glucose responses but associated with decreased insulin secretion (50). The result showed that Trp64Arg may affect IR by affecting insulin secretion rather than blood glucose level.

In summary, our study further clarified the relationship between Trp64Arg and IR and explained it at the gene level. It provides a direction for genetic research on the identification of therapeutic targets for clinical treatment of IR. However, the included studies are all case–control studies and cross-sectional studies; therefore, it is difficult to draw causal inferences. There may be selection bias in population selection, in either case–control studies or cross-sectional studies. And, measurement error may also exist. Finally, the number of articles included in our study is small, so more studies need to be included for further analysis.

## Conclusion

Our meta-analysis suggested that the Trp64Arg mutation in the beta-adrenergic receptor had a positive correlation with IR. Ethnicity, obesity status, blood sample source, and test status may be the potential factors affecting the relationship between Trp64Arg and IR.

## Data Availability Statement

The datasets presented in this study can be found in online repositories. The names of the repository/repositories and accession number(s) can be found in the article/**Supplementary Material**.

## Author Contributions

HW and CZ had the same contribution to the literature. HW and CZ designed the work, collected the data, conducted data analysis, and wrote the manuscript. ML, QL, QZ, DL, and Z-YM reviewed and edited the manuscript. JD had full access over all data in the study and is ultimately responsible for the decision to submit and publish the final version. All authors contributed to the article and approved the submitted version.

## Funding

This work was supported by two grants (to JD) from the National Natural Science Foundation of China (No. 31872791) and Natural Science Foundation of Shandong Province of China (No. ZR2019MC046).

## Conflict of Interest

The authors declare that the research was conducted in the absence of any commercial or financial relationships that could be construed as a potential conflict of interest.

## Publisher’s Note

All claims expressed in this article are solely those of the authors and do not necessarily represent those of their affiliated organizations, or those of the publisher, the editors and the reviewers. Any product that may be evaluated in this article, or claim that may be made by its manufacturer, is not guaranteed or endorsed by the publisher.

## Supplementary Material

Supplementary Figure 1 Impact analysis of individual studies. The study by E´ Erhardt had a significant effect on the pooled result.

Supplementary Figure 2 Forest plot after excluding a single study with high impact. 95% confidence intervals (*CI*) are expressed in bars (each group) and diamond (all studies). Summary estimates are analyzed using a random-effects model. SMD, standardized mean difference.

Supplementary Figure 3 Egger’s publication bias plot. *p*=0.796.

## References

[1] Brown AE Walker M. Genetics of Insulin Resistance and the Metabolic Syndrome. Curr Cardiol Rep (2016) 18:75. 10.1007/s11886-016-0755-4 27312935 PMC4911377

[2] Mauriège P Bouchard C. Trp64Arg Mutation in Beta 3-Adrenoceptor Gene of Doubtful Significance for Obesity and Insulin Resistance. Lancet (1996) 348:698–9. 10.1016/S0140-6736(05)65601-2 8806286

[3] Groop LC Bonadonna RC DelPrato S Ratheiser K Zyck K Ferrannini E. Glucose and Free Fatty Acid Metabolism in Non-Insulin-Dependent Diabetes Mellitus. Evidence for Multiple Sites of Insulin Resistance. J Clin Invest (1989) 84:205–13. 10.1172/JCI114142 PMC303971 2661589

[4] Kahn CR Neville DM Jr Roth J. Insulin-Receptor Interaction in the Obese-Hyperglycemic Mouse. A Model of Insulin Resistance. J Biol Chem (1973) 248:244–50. 10.1016/S0021-9258(19)44468-2 4348209

[5] Zhan S Ho SC. Meta-Analysis of the Association of the Trp64Arg Polymorphism in the Beta3 Adrenergic Receptor With Insulin Resistance. Obes Res (2005) 13:1709–19. 10.1038/oby.2005.209 16286518

[6] Lehtovirta M Kaprio J Forsblom J Eriksson J Tuomilehto L Groop L. Insulin Sensitivity and Insulin Secretion in Monozygotic and Dizygotic Twins. Diabetologia (2000) 43:285–93. 10.1007/s001250050046 10768089

[7] Leineweber K Büscher R Bruck H Brodde OE. Beta-Adrenoceptor Polymorphisms. Naunyn Schmiedebergs Arch Pharmacol (2004) 369:1–22. 10.1007/s00210-003-0824-2 14647973

[8] Artunc F Schleicher E Weigert C Fritsche A Stefan N Häring HU. The Impact of Insulin Resistance on the Kidney and Vasculature. Nat Rev Nephrol (2016) 12:721–37. 10.1038/nrneph.2016.145 27748389

[9] Emorine L Blin N Strosberg AD. The Human Beta 3-Adrenoceptor: The Search for a Physiological Function. Trends Pharmacol Sci (1994) 15:3–7. 10.1016/0165-6147(94)90118-X 8140656

[10] Widén E Lehto M Kanninen T Walston J Shuldiner AR Groop LC. Association of a Polymorphism in the Beta 3-Adrenergic-Receptor Gene With Features of the Insulin Resistance Syndrome in Finns. N Engl J Med (1995) 333:348–51. 10.1056/NEJM199508103330604 7609751

[11] Walston J Silver K Bogardus C Knowler WC Celi FS Austin S. Time of Onset of Non-Insulin-Dependent Diabetes Mellitus and Genetic Variation in the Beta 3-Adrenergic-Receptor Gene. N Engl J Med (1995) 333:343–7. 10.1056/NEJM199508103330603 7609750

[12] Clément K Vaisse C Manning BS Basdevant A Guy-Grand B Ruiz J. Genetic Variation in the Beta 3-Adrenergic Receptor and an Increased Capacity to Gain Weight in Patients With Morbid Obesity. N Engl J Med (1995) 333:352–4. 10.1056/NEJM199508103330605 7609752

[13] Fujisawa T Ikegami H Kawaguchi Y Ogihara T. Meta-Analysis of the Association of Trp64Arg Polymorphism of Beta 3-Adrenergic Receptor Gene With Body Mass Index. J Clin Endocrinol Metab (1998) 83:2441–4. 10.1210/jc.83.7.2441 9661625

[14] Allison D Heo M Faith M Pietrobelli AJIJOO. Meta-Analysis of the Association of the Trp64Arg Polymorphism in the β 3 Adrenergic Receptor With Body Mass Index. J Clin Endocrinol Metab (1998) 22:559–66. 10.1038/sj.ijo.0800625 9665677

[15] Kurokawa N Nakai K Kameo S Liu ZM Satoh G. Association of BMI With the Beta3-Adrenergic Receptor Gene Polymorphism in Japanese: Meta-Analysis. Obes Res (2001) 9:741–5. 10.1038/oby.2001.102 11743057

[16] Højlund K Christiansen C Bjørnsbo KS Poulsen P Bathum L Henriksen JE. Energy Expenditure, Body Composition and Insulin Response to Glucose in Male Twins Discordant for the Trp64Arg Polymorphism of the Beta3-Adrenergic Receptor Gene. Diabetes Obes Metab (2006) 8:322–30. 10.1111/j.1463-1326.2005.00509.x 16634992

[17] de Luis DA Aller R Izaola O Gonzalez Sagrado M Conde R. Relation of Trp64Arg Polymorphism of Beta 3-Adrenergic Receptor Gene to Adipocytokines and Fat Distribution in Obese Patients. Ann Nutr Metab (2008) 52:267–71. 10.1159/000144047 18617734

[18] De Luis Román DA Primo D Izaola O Aller R. Relation of Trp64Arg Polymorphism of Beta 3 Adrenoreceptor Gene With Metabolic Syndrome and Insulin Resistance in Obese Women. Nutr Hosp (2017) 34:383–8. 10.20960/nh.384 28421794

[19] Liberati A Altman DG Altman J Mulrow C Gotzsche PC Ioannidis JP. The PRISMA Statement for Reporting Systematic Reviews and Meta-Analyses of Studies That Evaluate Health Care Interventions: Explanation and Elaboration. J Clin Epidemiol (2009) 62:e1–34. 10.1016/j.jclinepi.2009.06.006 19631507

[20] Table. SydPath. (2017). Available at: https://www.who.int/news-room/fact-sheets/detail/e-coli. [Accessed March 5, 2021].

[21] Bindman AB. The Agency for Healthcare Research and Quality and the Development of a Learning Health Care System. JAMA Intern Med (2017) 177:909–10. 10.1001/jamainternmed.2017.2589 28542694

[22] Higgins JP Thompson SG. Quantifying Heterogeneity in a Meta-Analysis. Stat Med (2002) 21:1539–58. 10.1002/sim.1186 12111919

[23] Patsopoulos NA Evangelou E Ioannidis JP. Sensitivity of Between-Study Heterogeneity in Meta-Analysis: Proposed Metrics and Empirical Evaluation. Int J Epidemiol (2008) 37:1148–57. 10.1093/ije/dyn065 PMC6281381 18424475

[24] Piérola J Barceló A de la Peña M Barbé F Soriano JB Sánchez Armengo AI. Beta3-Adrenergic Receptor Trp64Arg Polymorphism and Increased Body Mass Index in Sleep Apnoea. Eur Respir J (2007) 30:743–7. 10.1183/09031936.00152006 17626108

[25] Pérez-Bravo F Echiburú B Maliqueo M Santos JL Sir-Petermann T. Tryptophan 64 –> Arginine Polymorphism of Beta-3-Adrenergic Receptor in Chilean Women With Polycystic Ovary Syndrome. Clin Endocrinol (Oxf) (2005) 62:126–31. 10.1111/j.1365-2265.2004.02183.x 15670186

[26] Erhardt E Czakó M Csernus K Molnár D Kosztolányi G. The Frequency of Trp64Arg Polymorphism of the Beta3-Adrenergic Receptor Gene in Healthy and Obese Hungarian Children and its Association With Cardiovascular Risk Factors. Eur J Clin Nutr (2005) 59:955–9. 10.1038/sj.ejcn.1602164 15942638

[27] de Luis DA Ballesteros M Ruiz E Muñoz C Penacho A Iglesias P. Polymorphism Trp64Arg of Beta 3 Adrenoreceptor Gene: Allelic Frequencies and Influence on Insulin Resistance in a Multicenter Study of Castilla-León. Nutr Hosp (2010) 25:299–303. 20449541

[28] de Luis DA Aller R Izaola O Gonzalez Sagrado M Conde R Castro MJ. Interaction of -55CT Polymorphism of UCP3 Gene With Trp64Arg Polymorphism of Beta3adrenoreceptor Gene on Insulin Resistance in Obese Patients. Eur Rev Med Pharmacol Sci (2012) 16:610–6. 22774401

[29] Dunajska K Lwow F Milewicz A Jedrzejuk D Laczmanski L Belowska-Bien K. Beta(3)-Adrenergic Receptor Polymorphism and Metabolic Syndrome in Postmenopausal Women. Gynecol Endocrinol (2008) 24:133–8. 10.1080/09513590801921686 18335327

[30] Mirrakhimov AE Kerimkulova AS Lunegova OS Moldokeeva CB Zalesskaya YV Abilova SS. An Association Between TRP64ARG Polymorphism of the B3 Adrenoreceptor Gene and Some Metabolic Disturbances. Cardiovasc Diabetol (2011) 10:89. 10.1186/1475-2840-10-89 21992420 PMC3215178

[31] Zawodniak-Szałapska M Stawerska R Brzeziańska E Pastuszak-Lewandoska D Lukamowicz J Cypryk K. Association of Trp64Arg Polymorphism of Beta3-Adrenergic Receptor With Insulin Resistance in Polish Children With Obesity. J Pediatr Endocrinol Metab (2008) 21:147–54. 10.1515/JPEM.2008.21.2.147 18422027

[32] Kuczmarski RJ Ogden CL Grummer-Strawn LM Flegal KM Johnson CLJAD. CDC Growth Charts: United States. (2000) 8:1–27. 11183293

[33] Yang LK Tao YX. Physiology and Pathophysiology of the Beta(3)-Adrenergic Receptor, in G Protein Signaling Pathways in Health and Disease. Tao YX, editor (2019), pp. 91–112. 10.1016/bs.pmbts.2018.09.003 30711031

[34] Strosberg AD. Association of Beta 3-Adrenoceptor Polymorphism With Obesity and Diabetes: Current Status. Trends Pharmacol Sci (1997) 18:449–54. 10.1016/S0165-6147(97)90681-7 9458691

[35] Yang L-K Tao Y-X. Physiology and Pathophysiology of the β3-Adrenergic Receptor. Prog Mol Biol Transl Sci (2019) 161:91–112. 10.1016/bs.pmbts.2018.09.003 30711031

[36] Lee YH. Meta-Analysis of Genetic Association Studies. Ann Lab Med (2015) 35:283–7. 10.3343/alm.2015.35.3.283 PMC4390695 25932435

[37] Adeva-Andany MM González-Lucán M Fernandez-Fernandez C Carneiro-Freire N Seco-Filgueira M Pedre-Pineiro AM. Diet Composition Determines Insulin Sensitivity and Cardiovascular Risk in Humans. Clin Nutr ESPEN (2019) 33:29–38. 10.1016/j.clnesp.2019.05.014 31451269

[38] Rivellese A De Natale C Lilli S. Type of Dietary Fat and Insulin Resistance. Ann N Y Acad Sci (2002) 967:329–35. 10.1111/j.1749-6632.2002.tb04288.x 12079860

[39] Rachek LI. Free Fatty Acids and Skeletal Muscle Insulin Resistance. Prog Mol Biol Transl Sci (2014) 121:267–92. 10.1016/B978-0-12-800101-1.00008-9 24373240

[40] Tai ES Tan ML Stevens RD Low YL Muehlbauer MJ Goh DL. Insulin Resistance Is Associated With a Metabolic Profile of Altered Protein Metabolism in Chinese and Asian-Indian Men. Diabetologia (2010) 53:757–67. 10.1007/s00125-009-1637-8 PMC3753085 20076942

[41] Würtz P Soininen P Kangas AJ Rönnemaa T Lehtimäki T Kähönen M. Branched-Chain and Aromatic Amino Acids are Predictors of Insulin Resistance in Young Adults. Diabetes Care (2013) 36:648–55. 10.2337/dc12-0895 PMC3579331 23129134

[42] Silver K Walston J Wang Y Dowse G Zimmet P Shuldiner AR. Molecular Scanning for Mutations in the Beta 3-Adrenergic Receptor Gene in Nauruans With Obesity and Noninsulin-Dependent Diabetes Mellitus. J Clin Endocrinol Metab (1996) 81:4155–8. 10.1210/jc.81.11.4155 8923875

[43] Shuldiner AR Sabra M. Trp64Arg Beta3-Adrenoceptor: When Does a Candidate Gene Become a Disease-Susceptibility Gene? Obes Res (2001) 9:806–9. 10.1038/oby.2001.109 11743064

[44] Barazzoni R Gortan Cappellari G Ragni M Nisoli E. Insulin Resistance in Obesity: An Overview of Fundamental Alterations. Eat Weight Disord (2018) 23:149–57. 10.1007/s40519-018-0481-6 29397563

[45] García-Rubi E Starling RD Tchernof A Matthews DE Walston JD Shuldiner AR. Trp64Arg Variant of the Beta3-Adrenoceptor and Insulin Resistance in Obese Postmenopausal Women. J Clin Endocrinol Metab (1998) 83:4002–5. 10.1210/jc.83.11.4002 9814483

[46] Urhammer SA Clausen JO Hansen T Pedersen O. Insulin Sensitivity and Body Weight Changes in Young White Carriers of the Codon 64 Amino Acid Polymorphism of the Beta 3-Adrenergic Receptor Gene. Diabetes (1996) 45:1115–20. 10.2337/diabetes.45.8.1115 8690160

[47] Jesus ÍC Alle LF Munhoz EC Silva LRD Lopes WA Tureck LV. Trp64Arg Polymorphism of the ADRB3 Gene Associated With Maximal Fat Oxidation and LDL-C Levels in Non-Obese Adolescents. J Pediatr (Rio J) (2018) 94:425–31. 10.1016/j.jped.2017.07.010 28941386

[48] Szendrei B González-Lamuño D Amigo T Wang G Pitsiladis Y Benito P. Influence of ADRB2 Gln27Glu and ADRB3 Trp64Arg Polymorphisms on Body Weight and Body Composition Changes After a Controlled Weight-Loss Intervention. Diabetes Obes Metab (2016) 41:307–14. 10.1139/apnm-2015-0425 26888112

[49] Højlund K Christiansen C Bjørnsbo KS Poulsen P Bathum L Henriksen JE. Energy Body Composition and Insulin Response to Glucose in Male Twins Discordant for the Trp64Arg Polymorphism of the β3 -Adrenergic Receptor Gene. Diabetes Obes Metab (2006) 8:322–30. 10.1111/j.1463-1326.2005.00509.x 16634992

[50] Christiansen C Poulsen P Beck-Nielsen H. The Trp64Arg Mutation of the Adrenergic Beta-3 Receptor Gene Impairs Insulin Secretion: A Twin Study. Diabetes Med (1999) 16:835–40. 10.1046/j.1464-5491.1999.00130.x 10547210

