# Supplementary figures and images for: The Association of Trp64Arg Polymorphism in the Beta-Adrenergic Receptor With Insulin Resistance: Meta-Analysis
Source: Front Endocrinol (Lausanne). 2021 Aug 26;12:708139. doi: 10.3389/fendo.2021.708139 (PMC8426512; doi:10.3389/fendo.2021.708139)

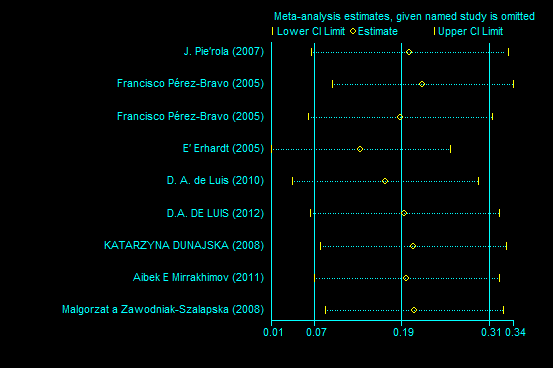

Supplement: Supplementary Figure 1 — Impact analysis of individual studies. The study by E´ Erhardt had a significant effect on the pooled result. [file Image_1.png]

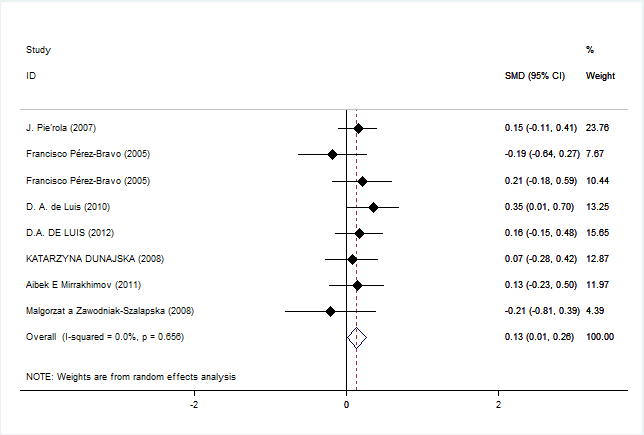

Supplement: Supplementary Figure 2 — Forest plot after excluding a single study with high impact. 95% confidence intervals (CI) are expressed in bars (each group) and diamond (all studies). Summary estimates are analyzed using a random-effects model. SMD, standardized mean difference. [file Image_2.tif]

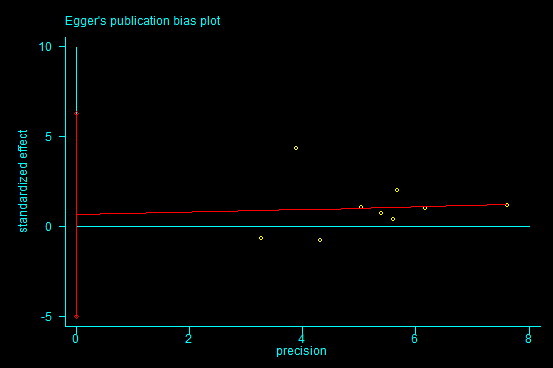

Supplement: Supplementary Figure 3 — Egger’s publication bias plot. p=0.796. [file Image_3.png]
